# How People with Lived Experiences of Substance Use Understand and Experience User Involvement in Substance Use Care: A Synthesis of Qualitative Studies

**DOI:** 10.3390/ijerph181910219

**Published:** 2021-09-28

**Authors:** Lillian Bruland Selseng, Brit-Marie Follevåg, Håvard Aaslund

**Affiliations:** 1 Faculty of Health and Social Sciences, Western Norway University of Applied Sciences, 6851 Sogndal, Norway; Brit.Marie.Follevag@hvl.no; 2 Faculty of Social Studies, VID Specialized University, 4024 Stavanger, Norway; Havaa@oslomet.no; 3 Faculty of Social Sciences, Oslo Metropolitan University, 0130 Oslo, Norway

**Keywords:** metasynthesis, substance use, substance use care, systematic review, user involvement, qualitative studies

## Abstract

There is a need for more knowledge on how people with substance use problems (SUPs) understand and experience user involvement when receiving care. In this systematic review, we identify and reanalyse the existing qualitative research that explores how people with lived experiences of substance use understand user involvement, and their experiences of key practices for achieving user involvement. We systematically searched seven electronic databases. We applied Noblit and Hare’s meta-ethnography, revised by Malterud, to identify, translate, and summarise the studies. The electronic search resulted in 2065 articles. We conducted a full-text evaluation of 63 articles, of which 12 articles met the inclusion criteria. The primary studies’ synthesis reveals three different understandings of user involvement: user involvement as joint meaning production, points of view represented, and user representation in welfare services. Key practices for achieving user involvement involved seeing and respecting the service user as a unique person, the quality of the interactional process, and the scope of action for people with SUPs, as well as professionals, including issues of stigma, power, and fatalism. The metasynthesis recognises the ambiguity of the concept of user involvement concept and the importance of including the service user’s perspective when defining user involvement. The analysis of key practices emphasises the importance of relational processes and contextual aspects when developing user involvement concepts.

## 1. Introduction

User involvement in substance use care is a fundamental goal of welfare services and has been emphasised in a number of strategies, plans, and declarations [1,2,3]. However, while user involvement is a crucial concept in literature concerning substance use problems (SUPs) and care, there are significant related challenges. First, measures related to the planning of substance use services are mainly derived from an expert perspective. Methods for including people with experiences of substance use in the planning of substance use services are weak and underdeveloped [1,4,5].

Second, the conceptualisation of user involvement is complex, variable, and multidimensional [1,2,3]. Tambuyzer et al. [3] found that a wide range of terms is used and that the understandings and practices related to user involvement varied considerably. Commonly used terms include shared decision making [6], peer engagement [5,7], consumer participation [1], involving patients [8], and patient-centred care [9,10]. The content of the concept also varies significantly, which has given rise to a wide range of ladders, cross tables, and typologies related to different groups of service users [11,12,13,14,15,16,17,18]. Such typologies have also been criticised for being one-dimensional and lacking the significant process orientation of user involvement [16,19]. Scholars have identified current user involvement practices as following a consumerist/managerialist tradition and a democratic/emancipatory tradition [19,20], as well as, potentially, a co-production tradition [21]. How user involvement is operationalised influences how user involvement research is conducted, how user involvement is received when put into practice, and how user involvement guidelines and requirements are designed. The understanding of user involvement will affect the situations one becomes concerned with, what one sees as a key condition, who one sees as a key actor, and what is seen as an outcome domain. The fact that the research shows that the perceptions of researchers and clinicians dominate means that it is also their perceptions that establish the grounds for what one is concerned with when user participation is to be investigated and improved.

Third, people with experiences of substance use have a different understanding of key concepts related to user involvement than researchers and health professionals. Bee et al.’s [22] systematic evidence synthesis of 117 studies suggests that user involvement fails because the patient’s frame of reference diverges from that of the providers. While the service users attributed the highest value to the relational aspects, the health professionals tended to define user involvement in terms of quantifiable outcomes. A comparison of the patient assessment of methadone maintenance treatment with the clinician-reported outcomes showed a significant divergence between patient-reported and clinician-reported improvement [23]. Based on a critical review and analysis of the literature on user satisfaction surveys in addiction treatment and harm reduction services, Trujols et al. [24] note that results of satisfaction surveys often diverge from results obtained via other data collection methods. Trujols et al. argue that this difference is related to conceptual confusion associated with terms, such as patient satisfaction and treatment satisfaction. These concepts are often treated as if clear, essential understandings of them exist, which is not the case. Based on their review, the researchers argue that the enthusiasm with user satisfaction surveys should be avoided and that a more participatory approach to programme evaluation is needed to reshape patient satisfaction surveys. They point out that the complex and nuanced experiences of service users can be more appropriately captured by qualitative approaches that utilise more generic, open-ended questions formulated in terms of service user experiences [24].

There is a need for more knowledge of how people with SUPs understand, define, and experience user involvement in substance use care. In order to fill this knowledge gap, we developed a study to explore and synthesise qualitative research on the perspectives of people with SUPs on user involvement. Meta-analyses of the included qualitative studies provide a systematic description and synthesis of the current knowledge base, which are essential for achieving the goal of user involvement in research and professional practice. In this systematic review, we identify and reanalyse the existing qualitative research that explores how people with lived experiences of substance use understand user involvement, and their experiences of key practices for achieving user involvement. The aim is to gain a thorough understanding of the perspectives of people with SUPs on user involvement.

## 2. Materials and Methods

We chose meta-ethnography as our method of analysis, which is a stepwise strategy for synthesising findings across qualitative studies [25], revised by Malterud [26]. The process includes seven steps: (1) getting started; (2) deciding what is relevant; (3) reading the studies; (4) determining how the studies are related; (5) translating the studies into one another; (6) synthesising translations; and (7) expressing the synthesis. We searched for studies in the following electronic databases: CINAHL (EBSCO), Embase, MEDLINE, PsycINFO (ProQuest), Scopus, SocINDEX, and Web of Science. Our searches used variations and combinations of terms that targeted five main concepts: service user perspectives, substance use problems, user involvement, substance use services, and qualitative research. Details of our search are presented in Table 1. Additionally, we performed a hand search using the reference list and citations of all identified studies selected for critical appraisal. We conducted the last search on 9 July 2021.

### 2.1. Data Collection

We exported the search results (2065 hits from the systematic search and 6 hits from the hand search) into the reference citation manager EndNoteX6 and removed duplicates (N-667). We then exported the references into the systematic review app Rayyan. Three independent reviewers (L.B.S., H.A., and B.M.F.) examined the titles and abstracts, and excluded irrelevant reports and papers using the inclusion and exclusion criteria (N-1340).

The included articles met the following criteria:

(1) The study was an empirical study, written in English, and published in a peer-reviewed journal;
(2) The study included qualitative data that explored the understandings and experiences of user involvement in substance use care from the perspective of people with lived experiences of substance use. In cases of mixed-method studies, only the qualitative results were included;
(3) The study sample consisted of people who were 18 years old or older, had lived experiences of substance use, and were receiving help from the welfare system to reduce any difficulties resulting from drug use;
(4) The study was published between January 2008 and June 2021.

**Figure 1:**
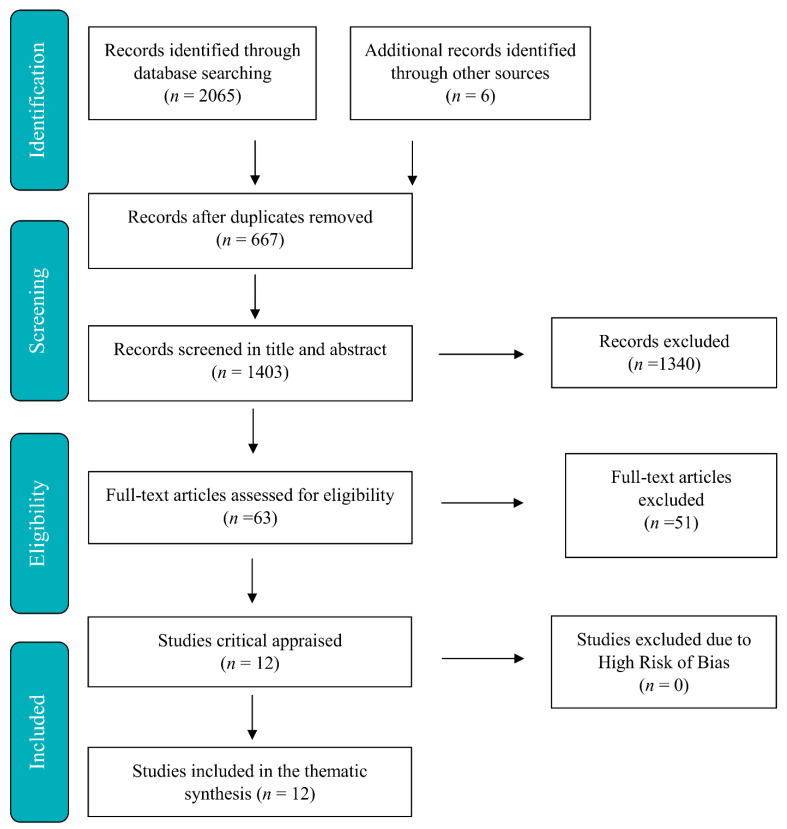
The PRISMA flow diagram.

Furthermore, any articles that did not focus specifically on user involvement were also excluded. All reviewers conducted a full-text screening of 63 publications, assessed their relevance, and excluded irrelevant papers (N-51). L.B.S., H.A., and B.M.F. evaluated the quality of the 12 eligible publications independently, and in accordance with the Critical Appraisal Skills Programme (CASP) checklist. Any disagreements in the screening and quality check process were resolved through discussions until a consensus was reached. An overview of the data collection can be seen in the flow diagram in Figure 1.

### 2.2. Characteristics of the Primary Studies

This meta-ethnography is based on findings from 12 primary studies conducted in Australia, Canada, Finland, Ireland, Norway, and the UK between 2008 and 2019 (Table 2). 

The studies included a total of 346 persons with experiences of substance use problems, with samples ranging from six to 83 informants. Although not all studies give details of the demographic variables of the participants, our impression is that they are broadly representative of age groups (18–69) and of male and female participants (Table 2). 

Seven studies were based solely on open-ended or semi-structured individual interviews, one study was based on focus groups, and four studies were based on combinations of these and other data sources, such as field notes. Six studies conducted thematic analyses, two were based on grounded theory, three used different variations of a participatory analysis, and one conducted a phenomenographic analysis. Six of the studies used the concept of service user involvement or user involvement, while the other six studies featured concepts, such as collaborative practice, consumer participation, empowerment/decision-making, peer engagement, stakeholder involvement, and the user’s choice/self-determination. Eight studies examined service user’s experiences being involved in their own drug treatment or substance use care. Three studies explored experiences from people engaged in user organisations providing services to others. One study examined drug users’ perspectives on peer engagement in health and harm reduction settings.

### 2.3. Analytic Methods

The results of these primary studies can be considered as the first-order analysis. Our analysis is a second-order analysis in which we synthesised the results of the first-order analysis. In line with Noblit and Hare [25], all authors read the articles thoroughly to find statements about user involvement, and identified relevant interpretative metaphors. We then ranked the articles in collaboration according to the amount of empirical data associated with our analytical focus. An index article was then chosen to act as a point of reference for comparing and interpreting other articles. The indexed article [2] contained detailed descriptions with illustrative metaphors, and was of a high methodological quality. We then listed the interpretive metaphors from each study vertically in separate columns of a grid in order to determine how the metaphors from the studies were connected. The vertical locations of the metaphors were adjusted so that each horizontal row contained thematically related metaphors.

Starting with the index article, we used Microsoft Excel to systematically code the content of the studies and to relate the studies to each other. The interpretation was idiomatic, and focused on the meaning content rather than the literal equivalents [25]. The studies were listed horizontally, and the encoded content was placed vertically in the grid, along with related content from other articles. Each article had two rows: one for interpretive metaphors and one for illustrative quotes or descriptions. We then synthesised the content by translating it into a new common concept: a reciprocal analysis [25]. Table 3 provides an excerpt from our second-order analysis for the first theme.

The reconceptualization achieved by the reciprocal analysis resulted in two categories: the fluid concepts of user involvement and key practices.

## 3. Results

### 3.1. The Fluid Concept of User Involvement

Three studies [2,27,28] explicitly explored how the informants understood user involvement. For the other studies, we examined the informants’ understandings of user involvement through the accounts they provided. The analysis showed that the studies presented a wide range of understanding of user involvement, and illustrated that user involvement is an unclear, multifaceted, and fluid concept. In the following paragraphs, we present the synthesis illustrated by selected quotations from the primary studies.

#### 3.1.1. User Involvement as Joint Meaning Production

It was evident in many of the primary studies that user involvement is understood as professionals engaging in meaning production, together with the user of the welfare services. In other words, user involvement was associated with the professionals arranging conversations with service users, wherein they gave them relevant information and assistance in reflecting upon what they wanted. User involvement understood as a joint meaning production may include decision making, but was not limited to making decisions. Fischer and Neale [29] and Fischer et al. [30] found that those who were new to treatment tended to report that they wanted and expected to be guided by staff, rather than to take the lead themselves. They perceived staff as being the experts who would know the best course of action to take. Hansen [31] pointed out that some informants expressed a form of ambivalence to the freedom of choice. Making choices was not always easy, and most of the participants appreciated the staff members as partners or co-producers of decisions. As a participant in Hansen’s study said, “I think they can decide a little too, I am not very good at deciding” [31] (p. 324). 

User involvement was associated with the informant’s subjective feeling that their humanity was recognised and acknowledge, and that their story was heard and respected [31,32,33]. Additionally, user involvement was related to interactions wherein the persons with lived experiences of substance use and the staff aiming to support them engaged in a trusting relationship that enabled knowledge to be inter-subjectively shared and developed through joint reflection work [31,32]. It was essential that service users felt that it was possible to discuss their problems with health professionals [31]. Patterson et al. [28] noted that user involvement was understood as a collaborative partnership, where service users’ expertise complemented formal management capabilities.

#### 3.1.2. User Involvement as a Point of View That Is Heard

Another understanding of user involvement evident in the primary studies was that the service users could make their own decisions and were equally involved in decisions about the service offer relevant to them. This understanding highlighted that people with lived experiences of substance use have knowledge that others do not have and, as a result, the best expertise [2]. Therefore, their views must be respected, taken seriously, and given a high level of validity [2,34]. The general public must be more aware of the issues that affect people with SUPs, and they as a group must have their voices heard [33]. The importance of gaining knowledge from lived experience of substance use is illustrated by these quotes from a male patient in the Norwegian public specialised mental health and substance abuse services: “The personnel must understand that their experience is not enough” [30] (p. 1236). A service user from England noted, “They can’t solve the problem without us” [35] (p. 59). A service user from Finland stated, “This is a kind of disease you can only comprehend if you have experience of your own. Nobody else understands what it means” [2] (p. 4).

In relation to service users having their views heard, some studies indicated that service users were in different places in the recovery process, which had implications for how much their opinions should be emphasised [30]. For example, Fischer et al. described how first-time service users expected to be guided by staff rather than to take the lead themselves, but had more to say as treatment progressed. 

#### 3.1.3. User Involvement as Representation in the Welfare Services

A third understanding of user involvement visible in the data material was the engagement of people with lived experiences of substance use to develop or participate in welfare services. The focus was on how they could use their knowledge and skills to deliver help and support services. Different variants of ex-users participating in the welfare system were highlighted, such as the employment of ex-drug users [29], and peer engagement in public health decisions, as well as the ex-user providing information to people having substance use problems [7], running drop-in offers [35], and being involved in the development, evaluation, and organising of services [2]. Van Hout and McElrath [36] referenced the Service User Support Team as a community employment scheme for those in recovery, which supports and advocates for people who use drugs and alcohol. Patterson et al. [35] describes how welfare service user groups provide forums for organisation-led consultation and user-agency communication. Service user groups also reported representation on decision-making bodies, and participated in agency training, recruiting agency staff, and quality assurance programmes [35]. It was emphasised in several of the studies that, due to their experience, people with lived experiences of substance use should be accorded a high level of validity when considering service design and delivery. This form of user involvement increased the quality of both the service offered by them and the service offered by the professional employees, and that their expertise was fundamental to effective service development [28,34].

### 3.2. Key Practices

We then examined the practices that were highlighted as being significant to user involvement. We investigated the different understandings of service user involvement to identify the key elements of a practice to be considered as involving service users, and the crucial components of a practice to not be considered as involving service users. This analysis resulted in three essential practices. A common feature of the primary studies was the identification of a gap between the ideals of user involvement and reality. The three essential practices were highlighted, both in terms of challenges in achieving user involvement and conditions for promoting user involvement. 

#### 3.2.1. To Been Seen and Respected as a Unique Person

A consistent feature of the primary studies was an emphasis on the importance of being seen, understood, respected, and met as a unique human being. This was expressed by service users through statements such as “having their ‘humanness’ recognised and acknowledged” [33] (p. 33); “listening and valuing” [2] (p. 4); “the issue of respect for service users was the key” [27] (p. 279); “respect and acceptance” [31]; “the importance of being listened to and met as fellow human beings” [32] (p. 133). In the words of one Norwegian boy: “It is crucial that I am met with respect and that the practitioner show that she cares about me, not seeing me as merely a ‘number in the system’” [32] (p. 132).

The importance of seeing the service user as a person and not as a category was relevant for user involvement at the individual and system levels. It was also relevant to how the professional was seen by the service user, and emphasised the importance of understanding how professionals thought and the reasoning behind their actions. This was expressed by a service user in Rance and Treloar’s [33] investigation of user involvement within Australian drug treatment services: “It’s good to know instead of, you know, seeing the staff as staff, you know, that they are people” [33] (p. 33). Similarly, the challenges that were highlighted in relation to achieving user involvement were linked to a lack of appreciation of subjectivity. These included challenges with “paternalist or negative attitudes of the staff” [2] (p. 6), stigma around drug users [7,28,31,33] (p. 231; p. 60; p. 322; p. 32); “[defining] service users in negative stereotypical terms” [27] (p. 282); and service users’ feelings of “being fixed or judged” [32] (p. 133); “being pathologised” [34] (p. 1235); staff “being negative or dismissive of them” [29] (p. 166); “fear of being outed” [7] (p. 232); and “staff being uninterested, unsympathetic or looking down on clients” [30] (p. 5).

#### 3.2.2. Quality of the Interaction Process

The quality of the interaction between people with lived experiences of substance use and the staff they met was fundamental to the feeling of being seen and respected as a unique person. The included studies presented a wide range of accounts and descriptions about the quality of the interactions. Descriptions such as “everyone’s working together” [33] (p. 33); “a place where everyone is heard” [33] (p. 33); and “joint reflection work” [31] (p. 323) all highlighted specific common positive characteristics. Ness et al. [32] pointed to a “collaborative practice” and described it as creating a “reflective space to make their own choices” (p. 135). Greer [7] described, “a non-judgemental and inclusive approach” (p. 233), while King [27] described a process that attempts to facilitate improved communication between service users and providers [27] (p. 279).

In studies that explored projects aiming to enhance user involvement, the characteristics used to describe the positive changes were closely linked to changes in how the participants interacted. Rance and Treloar [33] described it as a “different style of interaction” (p. 35), with “opportunities for both users and staff to come to know and ‘see’ one another better” (p. 34) and “creating new subject positions for both service-user and staff participants” (p. 35). Dimensions that were highlighted as significant for such an interaction were adequate and comprehensible information [2], well defined communication pathways [35], “improved communication between service providers and their clients” [29] (p. 161); “an opportunity to speak and be heard” [33] (p. 35); “time to listen and talk” [32] (p. 132); and “emergence of a more collaborative ethos of ‘working together’” [33] (p. 33). Furthermore, several of the primary studies emphasised that challenges related to different roles and power must be addressed [28,29,34,35].

Similarly, challenges related to user involvement were also related to challenges in the interaction between clients and staff. Patterson [28] noted a pervasive stigma and power imbalance, while Fischer et al. [30] highlighted negative staff behaviour that included staff being uninvolved, not listening to service users, and failing to follow through on their promises to the service users. In addition, Van Hout and McElrath [36] referenced power differentials, stigma, and one-way communication The analysis of the primary studies showed that it was essential for the welfare services to be able to arrange conversations that help both the client and the professional to understand more of each other and help them create a place for interaction and reflections [2,32,33,34].

#### 3.2.3. Scope of Action 

Another practice that was highlighted as necessary for user involvement was that the participants had a real room for manoeuvre. The need for flexibility applies both to the person with lived experience of substance use when meeting with professionals, and the professionals within their own system. The analysis of the primary studies showed that the participants appreciated floating and flexible services [31]. However, the analysis also indicated that opportunities for involvement were perceived as lacking. Rance and Treloar [33] found disenfranchisement in drug treatment, with service users expressing the opinion that “there’s no fucking point” [33] (p. 32). Laitila et al. [2] asserted that the “problems are that ‘systems make the rules’” (p. 5), and described the current, dominant division of power and knowledge, in which “the organisations were often hierarchical and inflexible” [2] (p. 5). Fischer and Neale [29] highlighted structural factors, such as limited resources, limited treatment availability, and lengthy waiting times, and Fischer et al. [30] noted that strict, arbitrary rules and procedures limited he service user’s room for manoeuvre, and angered them because they did not understand the rules and procedures or find them helpful. This annoyance could be counteracted by clearly explaining the rules’ rationale to them.

Larsen and Sagvaag [34] observed that “empowerment seemed to be perceived as something to be controlled and granted by leaders and staff” (p. 1238), which was illustrated by a patient’s comment that “it is possible to raise issues, but it has no impact” (p. 1236). Hansen [31] describe patients’ experiences of distrust and of risking punishment and exclusion if they did not do as recommended (p. 322). Patterson et al. argued that power relations were a key issue [35] (p. 92), which was also expressed in King [27] by a service user in Ireland: “I feel like I have to do what I’m told, like they have the power. We have no power. It’s like they have the key to your life” (p. 282). 

The need for room for manoeuvre was also linked to the fact that people with experience of SUPs find that they do not have the time or availability to engage in user involvement. Several studies [2,7,27,35,36] pointed out that informants expressed an inability to get involved while they were vulnerable themselves, as their own change process required so much of them. 

Several primary studies pointed to the significance of changing the scope of action in order to improve user involvement. Achieving increased freedom of choice for the service users and their helpers strengthened user involvement [29,34]. Furthermore, the value of flexible practitioners and services that provided opportunities for person-oriented service offerings was emphasised [31,32,34].

## 4. Discussion

The findings of this review raised two main topics: the fluid understanding of user involvement and key practices. The fluid understanding consisted of three different understandings of user involvement: joint meaning production, points of view, and representation in welfare services. Key practices included being seen and respected as a unique person, the quality of the interactional process, and the scope of action, including issues of stigma, power, and fatalism. The fact that our review points to ambiguous and fluid understandings of user involvement confirms previous studies that have described a lack of consensus on the definition of user involvement [1,2,3]. We will argue that the awareness that user involvement is understood in different ways has implications for how we can best facilitate the investigation and evaluation of user involvement and how we can best facilitate the promotion and evaluation of user involvement in drug treatment care. Hyshka et al. [4] conducted a scoping review to describe the extant research on the population’s need for substance use services and the extent to which such research incorporates expert and consumer perspectives on the population’s needs. They found that expert-driven approaches dominate the approaches to measuring the population’s needs. They indicate that the studies addressing consumer-defined need for substance use services are conceptually underdeveloped. Our review emphasises the importance of examining the service user’s perspective. Additionally, our review indicates the importance of not treating the user perspective as a single fixed view, but instead examining understandings of user involvement as being several, fluid, and contextually embedded. Furthermore, our review underscores the point made by Trujols et al. [24] that, in order to gain insight into service users’ perceptions of essential but ambiguous phenomena, such as user involvement and treatment satisfaction, it is important to have approaches that are open and exploratory and not based on a predetermined understanding of what user involvement is.

The key practices that we found point towards essential areas that should receive attention in research and clinical practice to improve user involvement. Many of the participants in the primary studies highlighted the gap between the ideals of user involvement and the actual outcomes in practice, and several of the studies that were included highlight the societal marginalisation of people with SUPs, the illegality of substance use, and the hierarchical and sometimes conspicuous tradition of many of the therapeutic approaches in the field. These might be regarded as significant institutional and cultural barriers to implementing a practice that addresses user involvement. Our findings also indicate that stigma is an essential theme. This is in line with Ti et al.’s [5] narrative literature review of studies assessing peer engagement in policy and programme development. They found that stigma and discrimination were crucial obstacles that peers face, and that future efforts should first focus on actively reducing issues of social stigmatisation. Moreover, Goodhew et al.’s [1] systematic review examining the activities and factors that facilitate consumer participation in drug treatment services indicates not only the negative attitudes of providers, but also power imbalances between consumers and providers, and a lack of consumer and organisational capacity that constrains consumer participation efforts. The fact that all of these reviews have similar implications is a powerful contribution to the awareness of what must be worked on for the sake of progressing user development in drug treatment care. Future research needs relate to addressing these obstacles and learning more about how to reduce obstacles such as stigma, power, and fatalism and to broaden the scope of action.

Our findings also indicate that there is a need for a dialogical turn and for a greater research focus on processes rather than a limited focus on specific decisions. The analysis of key practices points to the relevance of the conversations between the service user and the professional. Based on the review, we argue that these conversations themselves should be the subject of close examination and that it is important to see conversation as an important professional tool for enhancing user involvement.

For clinicians, the findings of this review should be promising, as the service users highlighted understandings and practices related to the professional role, values, and skills, which could feasibly be used to improve user involvement. However, the analysis also poses a significant challenge on a more systemic level to address the power differences and stigma within the services, and enhance the possibilities for staff to manoeuvre in ways that make involvement worth the effort, and organise the services accordingly.

### Strength and Limitations

The strengths of this study are evident in its transparency of the study, as the inclusion of studies was determined through a detailed and transparent analytical approach by three independent researchers, and the review was conducted according to PRISMA guidelines [37]. However, while this is the first systematic review concerning the experience of people with SUPs of user involvement, there are significant limitations to our findings. Our search strategy cannot guarantee full coverage of the eligible primary studies. Furthermore, the different conceptualisations of user involvement and different understandings result in different approaches to the phenomenon, which has affected our findings. For example, it is not always clear whether the understanding of user involvement comes from the researcher or the participant. In addition, the practitioners in welfare services—whether included in the study or not—will also affect the concept of user involvement. In line with our finding of user involvement as contextually embedded understanding, it should also be noted that the studies included represent quite different types of services and institutional contexts. As previously mentioned, the area of user involvement studied in the primary studies also differs from one’s own treatment or care, services for others, and peer engagement. Consequently, these differences will also affect the understandings of user involvement, practices and scope of action. In addition, our sample seems skewed, as it only represents English speaking and Scandinavian countries, but it is unclear whether this corresponds to a skewed interest in user involvement in practice or in research (or both), and which other biases could be hidden in the sample. In general, it has been difficult to determine which persons with lived experiences of substance use are represented in the sample, as regards intersectional categories such as class, race and gender, as well as the length and type of substance use, such as people in recovery vs people who use drugs.

## 5. Conclusions

The results of this metasynthesis provide a consolidated picture of three different understandings of user involvement. These results suggest that while the term “user involvement” is commonly used, it is a fluid and ambiguous concept. Since understandings of the concept have consequences for expectations of user involvement, practices, and focus areas, these findings have implications for improving both the research on user involvement and future clinical practice to ensure user involvement. Due to the ambiguity of the concept, the analysis highlights the importance of continuously examining how user involvement is understood and including the service users’ perspectives when investigating and defining user involvement. Furthermore, the study synthesises key practices due to user involvement. The metasynthesis underscores that there are significant shortcomings linked to the realisation of user involvement, and the findings suggest a focus on relational and contextual factors as stigma, power and fatalism to encourage user involvement.

## Figures and Tables

**Table 1:** Concepts and terms in the search string.

Concepts	Terms
Concept I: service user perspectives	“user participat*” OR rights OR cooperation OR co?production OR involvement OR participation OR engagement OR “take part” OR patient satisfaction OR patient?centered evaluation OR service user perspective OR patient perception OR shared decision-making.
Concept II: substance use problems	“drug-related” OR “drug abuse*” OR “drug misuse*” OR “illicit drug*” OR “substance abuse*” OR “problematic substance use” OR addiction.
Concept III: user involvement	participation OR involvement OR user involvement OR engag* OR patient activat* OR empowerment OR shared decision* OR partnership OR consumer participation OR experience* OR opinion* OR view* OR meaning* OR knowledge* OR understand* OR attitude OR satisfaction OR voices.
Concept IV: substance use services	“social service” OR support* OR counsel* OR “social work” OR practitioner OR “health service” OR therap* OR “mental health” OR “social care” OR “local service” OR “welfare service” OR postvention OR treatment OR harm reduction service OR health care OR substance use services OR drug treatment service.
Concept V: qualitative research	focus group* OR qualitative research OR qualitative study OR qualitative studies OR qualitative method* OR interviews OR phenomenolog* OR interpretive OR interpretative OR hermeneutic* OR “first person” OR “self-report*” OR narrativ* OR “grounded theory” OR “field stud*”

*: The use of the asterisk (*) is a searching technique used in several databases called truncation, and it is used to replace a word ending. It enables different forms of a word to search for simultaneously and will increase the number of search results found.

**Table 2:** Presentation of included contributions and key features.

Article	Country	Data	Theoretical Concept	Relevant Sample	Analytical Approach	Research Question/Aim
Fischer, J., Neale, J., Bloor, M., & Jenkins, N. (2008). Conflict and user involvement in drug misuse treatment decision-making: a qualitative study. Substance abuse treatment, prevention, and policy, 3(1), 21.	UK	Individual interviews	User involvement	79 clients in residential and community drug treatment agencies; 53 men and 26 women.	Thematic analysis	Develop our understanding of user involvement by examining the extent, causes, responses to, and outcomes of conflicts occurring between the clients and staff of drug treatment services.
Fischer, J., & Neale, J. (2008). Involving drug users in treatment decisions: An exploration of potential problems. Drugs: education, prevention and policy, 15(2), 161–175.	UK	Individual interviews	User involvement	Same as the above.	Thematic analysis	To investigate the problems that might specifically arise when involving drug users in making their ‘own’ treatment decisions.
Greer, A. M., Amlani, A., Burmeister, C., Scott, A., Newman, C., Lampkin, H., … & Buxton, J.A. (2019). Peer engagement barriers and enablers: insights from people who use drugs in British Columbia. Canadian Journal of Public Health 110(2), 227–235	Canada	Peer-facilitated focus groups	Peer engagement	83 participants who used illicit drugs, aged 18–64; 38 males, 30 females, 2 trans, and 13 unknown.	Thematic analysis, participatory coding	Examining the perspectives of people who use or have used illicit drugs (PWUD) on peer engagement in health and harm reduction settings across British Columbia (BC), Canada.
Hansen, I. L. S. (2018). Users’ choice in providing services to the most vulnerable homeless people. Social Inclusion, 6(3), 319–326.	Norway	Individual interviews	User’s choice/ self-determination	16 participants in Housing First with severe substance use problems and mental illness; 13 men and 3 women, 9 aged 30–49, 6 over 50, and 1 under 30.	Thematic analysis	Discuss users’ experiences from participating in Housing First programs in Norway (provide a broad range of services, including various forms of practical assistance, help with personal finances, counselling, help establishing and maintaining contact with other social and health services, and coordination of service provision on an individual basis).
King, A. (2011). Service user involvement in methadone maintenance programmes: The ‘philosophy, the ideal and the reality’. Drugs: education, prevention and policy, 18(4), 276–284.	Ireland	Individual interviews	Service user involvement	8 service users from different clinics, aged over 18.	Thematic content analysis	To explore user involvement processes within methadone maintenance programs, aiming to establish the degree to which partnership and collaboration exists between the users and providers of Irish drug treatment services.
Larsen, T., & Sagvaag, H. (2018). Empowerment and pathologization: A case study in Norwegian mental health and substance abuse services. Health Expectations, 21(6), 1231–1240.	Norway	Minutes, dialogue meetings, multistage focus group, individual interviews	Empowerment/decision-making	6 patients in opioid maintenance treatment, (+ 5 co-researchers).	Content analysis, member-checking	To explore what may hinder patients’ voices being heard when collaborating with staff and leaders to improve services.
Laitila, M., Nikkonen, M., & Pietilä, A. M. (2011). Involvement in mental health and substance abuse work: conceptions of service users. Nursing research and practice, 2011.	Finland	Individual interviews	Service user involvement	27 service users with experience from mental health and/or substance abuse services. (Snowball sample); 10 women and 17 men, 9 aged 31–50, 5 under 30, and 4 over 50.	Phenomenographic analysis	What are service users’ conceptions of SUI in mental health and substance abuse work?
Ness, O., Kvello, Ø., Borg, M., Semb, R., & Davidson, L. (2017). “Sorting things out together”: Young adults’ experiences of collaborative practices in mental health and substance use care. American Journal of Psychiatric Rehabilitation, 20(2), 126–142.	Norway	Individual interviews	Collaborative practice	7 service users, 2 females and 5 males aged 20–30.	Thematic analysis	How do young adult service users with co-occurring mental health and substance use problems understand and describe collaborative practice with community mental health practitioners?
Patterson, S., Weaver, T., & Crawford, M. (2010). Drug service user groups: Only a partial solution to the problem of developing user involvement. Drugs: education, prevention and policy, 17(1), 84–97.	UK	Focus group interviews	User involvement	78 participants in “user groups” aged 20 to 50, approximately 1/3 female.	Grounded theory	To investigate and describe the role of drug service user groups in local service user involvement (UI).
Patterson, S., Weaver, T., Agath, K., Albert, E., Rhodes, T., Rutter, D., & Crawford, M. (2009). ‘They can’t solve the problem without us’: a qualitative study of stakeholder perspectives on user involvement in drug treatment services in England. Health & social care in the community, 17(1), 54–62.	UK	Focus group interviews and individual interviews	User involvement	Same as the above.	Grounded theory	To develop a contextualised description of UI in drug treatment based upon an analysis of the experiences and perspectives of those commissioning, managing, providing and using services.
Rance, J., & Treloar, C. (2015). “We are people too”: Consumer participation and the potential transformation of therapeutic relations within drug treatment. International Journal of Drug Policy, 26(1), 30–36.	Australia	Individual interviews	Consumer participation	30 consumers in three drug treatment facilities undergoing a participation project, aged 25–69.	Adaptive coding	What made such transformation possible?
Van Hout, M. C., & McElrath, K. (2012). Service user involvement in drug treatment programmes: Barriers to implementation and potential benefits for client recovery. Drugs: Education, Prevention and Policy, 19(6), 474–483.	Ireland/ UK	Individual interviews and field notes	Service user involvement	12 service users from organizations providing services to individuals experiencing problem drug misuse. Age 20–60, reported 9 females and 2 males (!)	Thematic analysis	To investigate user and treatment provider perspectives of the nature and extent of service user involvement in the region, and explore the perceived benefits and limitations of implementing service user forum(s) in the region.

**Table 3:** Example of grid for reciprocal translation; content issues from the primary articles about understandings and experiences of user involvement.

Hansen (2018)	Statements from Informants	Rance and Treloar (2015)	Statements from Informants	Næss et al. (2017)	Statements from Informants	Our Translation	Second-Order Analyses
Respect and acceptance	They have to listen to me! They see behind your behaviours, see the person in this. You feel worthy when you meet them, that you are of importance.	Recognized and acknowledged humanness, subjectivity	They can see that you know, you are human	Don’t fix me or judge me	It is crucial that I am met with respect and that the practitioner show that she care about me, not seeing me as merely a “number in the system.”	To be respected and recognized as a subject	User involvement as joint meaning-production
Joint reflection work (The analysis shows that the staff contribute with advice, guidance, questions, and opinions.)		Collaborative ethos	Everyone working together	Trusted partner to make stimulating reflections.	If the practitioners don’t know me, and they don’t ask me questions, but just give me advice or tell me what to do, that always make me do the opposite.	Joint meaning–production	
Engaging in making choices is not always easy-appreciate the staff as partners or co-producers of decisions.	I think they can decide a little too, I am not very good at deciding.	Interaction	Enhanced opportunities for interaction	Someone to sort issues out with	Having good conversations with the practitioners helped me to clear my mind. If this didn’t happen, I just got stuck and overwhelmed with everything.	User involvement is about shared decisions	

## Data Availability

All included studies are shown in Table 2.
